# Supplementary material for: The role of precursor coverage in the synthesis and substrate transfer of graphene nanoribbons
Source: Nanoscale Adv. 2025 Feb 11;7(7):1962–71. doi: 10.1039/d5na00017c (PMC11812448; doi:10.1039/d5na00017c)
Supplement: NA-007-D5NA00017C-s001 [file NA-007-D5NA00017C-s001.pdf]

Supplementary Information (SI) for Nanoscale Advances.
This journal is © The Royal Society of Chemistry 2025

SUPPORTING INFORMATION

The role of precursor coverage in the synthesis and substrate transfer of graphene nanoribbons

Rimah Darawish[1,2], Oliver Braun[3,4], Klaus Müllen[5,6], Michel Calame[3,4,7], Pascal Ruffieux[1], Roman Fasel[1,2] and Gabriela Borin Barin[1]*

[1]Empa, Swiss Federal Laboratories for Materials Science and Technology, nanotech@surfaces Laboratory, 8600 Dübendorf, Switzerland

[2]Department of Chemistry, Biochemistry and Pharmaceutical Sciences, University of Bern, 3012 Bern, Switzerland

[3]Empa, Swiss Federal Laboratories for Materials Science and Technology, Transport at Nanoscale Interfaces Laboratory, 8600 Dübendorf, Switzerland

[4]Department of Physics, University of Basel, 4056 Basel, Switzerland

[5]Max Planck Institute for Polymer Research, 55128 Mainz, Germany

[6]Department of Chemistry, Johannes Gutenberg University Mainz, Duesbergweg 10-14, 55128, Mainz, Germany

[7]Swiss Nanoscience Institute, University of Basel, 4056 Basel, Switzerland

*corresponding author: gabriela.borin-barin@empa.ch

Length evolution of 9-AGNRs on Au(788)

Table S1. Length evolution of 9-AGNRs on Au(788). Average length (in nm) of GNRs at the 1st-, 2nd-, and 3rd-row positions for 30 samples of 9-AGNRs with different PDs ranging from 1-9 Å, along with their respective standard deviation.

Precursor dose	1Å	2Å	3Å	4Å	5Å	6Å	7Å	8-9Å
GNR length at 1st-row position	14± 2	19± 5	35± 4	39± 4	43± 3	44± 2	45± 2	46± 2
GNR length at 2nd-row position				14± 5	22± 3	30± 2	36± 3	42± 5
GNR length at 3rd-row position							13± 2	36±5

9-AGNR quality and alignment as a function of PD on Au(788) and after substrate transfer to ROS

Table S2. Average peak position (in cm^{-1}) of RBLM, CH, D, and G modes measured for PDs from 1-9 Å in samples on Au(788) (40 samples) and after substrate transfer onto ROS (27 samples). Data are obtained from Raman maps acquired in a vacuum chamber using a 785 nm excitation wavelength.

Raman mode	RBLM		CH		D		G	
Substrate	Au(788)	ROS	Au(788)	ROS	Au(788)	ROS	Au(788)	ROS
1 Å	311±1	313±1	1242±3	1231±1	1334±1	1337±1	1596±1	1593±1
2 Å	311±1	312±1	1245±3	1235±2	1335±2	1337±1	1596±1	1593±2
3 Å	312±1	313±1	1245±2	1235±1	1336±1	1338±1	1597±1	1593±2
4 Å	312±2	313±1	1242±4	1235±1	1334±3	1337±1	1597±1	1594±2
5 Å	311±1	314±1	1246±2	1234±2	1334±1	1337±1	1596±1	1594±1
6 Å	311±1	313±1	1247±2	1236±2	1336±2	1338±1	1596±1	1594±2
7 Å	311±1	312±1	1247±3	1235±2	1338±2	1339±1	1597±1	1595±2
8-9 Å	312±1	313±1	1245±2	1232±2	1339±2	1339±1	1597±1	1594±1

Table S3. Average full-width at half maximum (FWHM, in cm^{-1}) of RBLM, CH, D, and G modes measured for PDs from 1-9 Å in samples on Au(788) (40 samples) and after substrate transfer onto ROS (27 samples). Data are obtained from Raman maps acquired in vacuum using a 785 nm excitation wavelength.

Raman mode	RBLM		CH		D		G	
Substrate	Au(788)	ROS	Au(788)	ROS	Au(788)	ROS	Au(788)	ROS
1 Å	14±1	15±2	30±5	35±4	13±2	14±1	11±2	13±1
2 Å	13±1	16±2	30±6	38±6	14±2	15±1	12±2	12±1
3 Å	15±2	17±5	29±5	33±9	13±2	19±8	13±2	12±1
4 Å	13±1	15±3	32±6	37±4	14±2	17±5	13±2	14±2
5 Å	14±1	16±1	32±5	33±4	13±1	16±4	12±2	14±1
6 Å	14±1	16±1	32±5	33±4	15±1	16±4	13±1	14±1
7 Å	16±1	15±1	33±5	32±4	17±2	18±4	14±2	15±2
8-9 Å	16±1	15±2	32±4	32±4	17±2	18±4	14±2	15±2

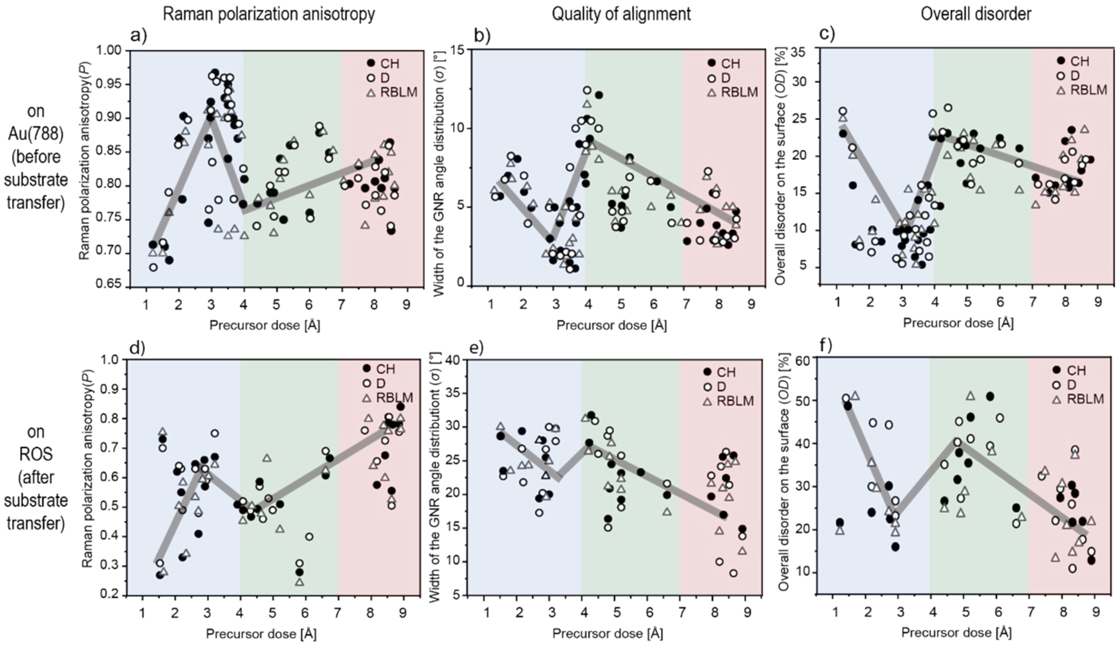

Figure S1: Impact of PD on the alignment of 9-AGNRs on Au(788) and after substrate transfer onto ROS. The alignment was evaluated using Eq (2) from polarized Raman data for RBLM, CH, and D modes. Panels (a), (b), and (c) show the average *P, σ,* and *OD* of RBLM, CH, and D modes, respectively, as a function of PD from 1-9 Å on Au(788) and panels (d), (e) and (f) after substrate transfer onto ROS. The colored background areas represent the growth regimes in which GNRs predominantly grow at the 1st-row position (blue), at the 2nd-row position (green), and at the 3rd-row position (red) and the grey lines are guides to the eye. The data are acquired in a vacuum using 785 nm excitation.

The impact of inefficient substrate transfer on GNR integrity and alignment

Here, we investigate the effect of inefficient substrate transfer on the integrity and alignment of GNRs in low-PD samples (3 Å). Figure S2 shows the Raman spectra and polar plot for a sample with PD = 3 Å on the growth surface Au(788) and after inefficient substrate transfer onto ROS. Table S4 presents the FWHM and peak position for the RBLM, CH, D, and G modes acquired with the 9-AGNRs on Au(788) and after their transfer onto ROS. Our observations indicate that inefficient substrate transfer resulted in increased FWHM values and peak shifts for all active Raman modes.

Specifically, we observe a large shift of -10 cm^{-1} for the RBLM and a significant shift of +44 cm^{-1} for the CH mode. The FWHM of RBLM, CH, D, and G modes broaden from 11, 31, 12, and 13 cm^{-1} on Au(788) to 25, 100, 85, and 44 cm^{-1} on ROS, respectively, confirming the inefficiency of the substrate transfer for this sample. Changes in the CH-D region can be attributed to damage in the GNR edge structure[1–3], while changes in the G and RBLM modes suggest the presence of defects and/or doping[4–6].

To investigate the influence of inefficient substrate transfer on the alignment and overall disorder on the surface (*OD*), we extract *P*, *σ*, and *OD* on both substrates for the G mode (Table S5). We observe a significant decrease in *P* along with broadening of *σ*: $P_{Au(788)}$= 0.92 ($\sigma_{Au(788)}$= 1°) to P_{ROS}= 0.40 (σ_{ROS}= 40°). Additionally, *OD* increases from 14% on Au (788) to 35% on ROS. These findings confirm that GNRs are susceptible to defects and can vary in quality upon electrochemical delamination transfer.

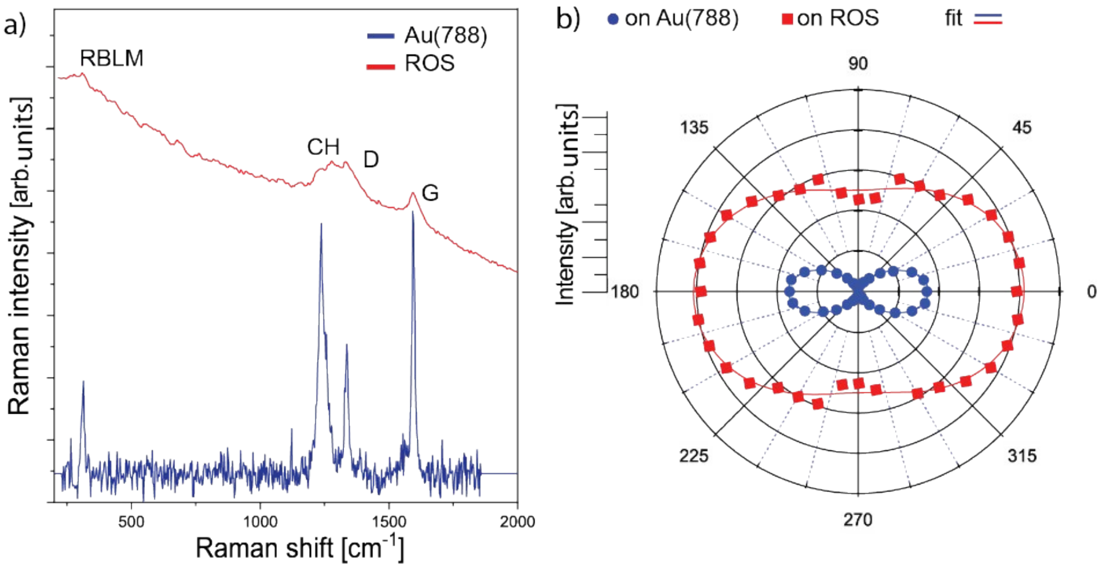

Figure S2. Raman spectra and G-mode polar plots for a 9-AGNR sample grown with PD = 3 Å on Au(788) and after inefficient substrate transfer onto the ROS. (a) Raman spectra on Au(788) in blue and on the ROS in red. (b) Polar plot of the Raman intensity of the G-mode on Au(788) in blue and on the ROS in red. Spectra were acquired using an excitation wavelength of 785 nm under vacuum conditions and changing the incident laser's angle (θ_{in}) with respect to GNRs' alignment direction (θ = 0°), without utilizing a polarizer in the detection path.

Table S4. Peak position and FWHM of the RBLM, CH, D, and G modes for the sample in Fig. S2 on Au(788), and after transfer onto ROS.

Substrate	Raman mode	RBLM	CH	D	G
Au(788)	Peak position [cm^{-1}]	312	1240	1339	1593
	FWHM [cm^{-1}]	11	31	12	13
ROS	Peak position [cm^{-1}]	302	1284	1337	1591
	FWHM [cm^{-1}]	25	100	85	44

Table S5. *P, σ*, and *OD* of the G mode for the sample in Fig. S2 on Au(788), and after transfer onto ROS.

Substrate	Au(788)	ROS
Raman polarization anisotropy (*P*)	0.92	0.4
Quality of alignment (*σ*) [°]	1±0.05	40±0.8
Overall disorder on the surface (*OD*) [%]	14	35

References

(1) Senkovskiy, B. V.; Pfeiffer, M.; Alavi, S. K.; Bliesener, A.; Zhu, J.; Michel, S.; Fedorov, A. V.; German, R.; Hertel, D.; Haberer, D.; Petaccia, L.; Fischer, F. R.; Meerholz, K.; van Loosdrecht, P. H. M.; Lindfors, K.; Grüneis, A. Making Graphene Nanoribbons Photoluminescent. *Nano Lett.* **2017**, *17* (7), 4029–4037. https://doi.org/10.1021/acs.nanolett.7b00147.

(2) Barin, G. B.; Fairbrother, A.; Rotach, L.; Bayle, M.; Paillet, M.; Liang, L.; Meunier, V.; Hauert, R.; Dumslaff, T.; Narita, A.; Müllen, K.; Sahabudeen, H.; Berger, R.; Feng, X.; Fasel, R.; Ruffieux, P. Surface-Synthesized Graphene Nanoribbons for Room-Temperature Switching Devices: Substrate Transfer and Ex-Situ Characterization. *ACS Appl. Nano Mater.* **2019**, *2* (4), 2184–2192. https://doi.org/10.1021/acsanm.9b00151.

(3) Overbeck, J.; Borin Barin, G.; Daniels, C.; Perrin, M. L.; Liang, L.; Braun, O.; Darawish, R.; Burkhardt, B.; Dumslaff, T.; Wang, X.-Y.; Narita, A.; Müllen, K.; Meunier, V.; Fasel, R.; Calame, M.; Ruffieux, P. Optimized Substrates and

Measurement Approaches for Raman Spectroscopy of Graphene Nanoribbons. *Phys. Status Solidi B* **2019**, *256* (12), 1900343. https://doi.org/10.1002/pssb.201900343.

(4) Senkovskiy, B. V.; Usachov, D. Yu.; Fedorov, A. V.; Marangoni, T.; Haberer, D.; Tresca, C.; Profeta, G.; Caciuc, V.; Tsukamoto, S.; Atodiresei, N.; Ehlen, N.; Chen, C.; Avila, J.; Asensio, M. C.; Varykhalov, A. Yu.; Nefedov, A.; Wöll, C.; Kim, T. K.; Hoesch, M.; Fischer, F. R.; Grüneis, A. Boron-Doped Graphene Nanoribbons: Electronic Structure and Raman Fingerprint. *ACS Nano* **2018**, *12* (8), 7571–7582. https://doi.org/10.1021/acsnano.8b04125.

(5) Ma, C.; Xiao, Z.; Puretzky, A. A.; Baddorf, A. P.; Lu, W.; Hong, K.; Bernholc, J.; Li, A.-P. Oxidization Stability of Atomically Precise Graphene Nanoribbons. *Phys. Rev. Mater.* **2018**, *2* (1), 014006. https://doi.org/10.1103/PhysRevMaterials.2.014006.

(6) Senkovskiy, B. V.; Fedorov, A. V.; Haberer, D.; Farjam, M.; Simonov, K. A.; Preobrajenski, A. B.; Mårtensson, N.; Atodiresei, N.; Caciuc, V.; Blügel, S.; Rosch, A.; Verbitskiy, N. I.; Hell, M.; Evtushinsky, D. V.; German, R.; Marangoni, T.; van Loosdrecht, P. H. M.; Fischer, F. R.; Grüneis, A. Semiconductor-to-Metal Transition and Quasiparticle Renormalization in Doped Graphene Nanoribbons. *Adv. Electron. Mater.* **2017**, *3* (4), 1600490. https://doi.org/10.1002/aelm.201600490.
